# Identification and validation of genomic regions for pod shatter resistance in *Brassica rapa* using QTL-seq and traditional QTL mapping

**DOI:** 10.1186/s12870-025-06155-z

**Published:** 2025-02-10

**Authors:** Rosy Raman, Yu Qiu, N. Coombes, Harsh Raman

**Affiliations:** NSW Department of Primary Industries and Rural Development, Wagga Wagga Agricultural Institute, Wagga Wagga, NSW 2650 Australia

**Keywords:** *Brassica rapa*, Pod shatter resistance, QTL-seq, Linkage mapping, Dehiscence zone formation

## Abstract

**Background:**

Pod shatter resistance is an important trait in Brassica species, significantly impacting the yield and profitability of growers. Identifying genomic regions and understanding genes underlying shatter resistance is a major objective of breeding programs. *Brassica rapa*, commonly known as rape or field mustard, is an ancestral species of *Brassica napus* and *Brassica juncea* – the most widely oilseed crops grown worldwide. In this study, we performed diversity analysis of *B. rapa* accessions, bulked segregant analysis based quantitative trait locus-sequencing (QTL-seq), and traditional quantitative trait locus (QTL) mapping in an F_2_ population to identify genomic regions associated with pod shatter resistance in *B. rapa*.

**Results:**

A considerable genetic variation for pod shatter resistance, measured as rupture energy (RE), varied from 0.63 to 3.49 mJ^(½)^ was revealed among 90 accessions of *B. rapa*. Cluster analysis based on 10,324 DArTseq markers showed that pod shatter-resistant accessions originated from diverse sources. We further investigated the genetic and anatomical bases of variation in pod shatter resistance from two contrasting parental lines, ATC90153 (maternal parent with high RE) and ATC91215 (paternal parent with low RE). Bulked segregant resequencing analysis of parental lines and two pooled samples, prepared from 10 resistant and 10 sensitive lines to pod shatter, identified three genomic regions for shatter resistance on chromosomes A06 and A09. Traditional QTL analysis validated marker-pod shatter resistance associations on chromosomes A06 and A09 in the same F_2_ population using a linkage map based on 23,274 DArTseq markers. Physical positions of significantly associated markers and the *priori* pod dehiscence genes on the *B. rapa* reference genome sequence suggested *BEE1/PEROXIDASE/TCP8* on A06 and *ADPG1/SHP1/MYB116* genes on A09 as potential candidates for pod shatter resistance. Sequence comparison of parental lines identified sequence variants (194 SNPs and 74 InDELs on A06, and two SNPs and two InDELs on A09) in the promoter and downstream regions of *B. rapa* genes within the QTL.

**Conclusions:**

We identified QTLs and *priori* candidate genes associated with variation in pod shatter resistance on chromosomes A06 and A09 in *B. rapa*. This study provides potential gene targets to understand molecular mechanisms and improve pod shatter resistance in Brassica crops.

**Supplementary Information:**

The online version contains supplementary material available at 10.1186/s12870-025-06155-z.

## Background

Deciphering the genetic basis of trait variation is vital to understanding the nature of inheritance and the transmission of alleles into the progenies for the accelerated genetic improvement of crops, required to meet the global energy demands for food, feed, fuel, and fibre. Current breeding programs are making genetic progress by moving from traditional breeding based on exclusive phenotypic selection to new breeding technologies based on both phenotypic and genotypic selection for agronomically important traits [1–3]. The latter has been possible by implementing innovative high-throughput genotyping, genome sequencing, trait-marker analytics, and phenotypic/phenomics and genomic selection tools [4–6].

Genetic resistance to pod shattering is a critical trait that ensures minimal seed loss during windrowing and mechanical harvesting. This domestication-related trait has been selected for seed production in several broadacre crops. However, the majority of cultivated oilseed brassica crops such as *B. rapa* (AA, 2*n* = 2× = 20), *Brassica napus* (AACC genome, 2*n* = 4×= 38), and *Brassica juncea* (AABB genome, 2*n* = 4×=36) [7] are highly prone to shattering. Brassica growers seek cultivars with pod shatter resistance, as shattering can result in yield losses of up to 70% under unfavourable conditions [8] and underpin growers’ profitability on the return of their investment. Pod shatter-prone cultivars release and scatter seeds on the ground, which become weeds in a subsequent crop if not managed adequately with agronomic interventions. At physiological maturity, the seeds enclosed in pods naturally dehisce due to the formation of a dehiscence zone (DZ) between the valve and replum in many crops, including members of Brassicaceae and Fabaceae [9–12]. DZ forms due to the tensile forces, water loss at maturity and action of hydrolytic enzymes in the pods [9, 13–15].

*Brassica rapa*, commonly known as rape or field mustard/turnip, belongs to the Brassicaceae family, which includes the model plant, Arabidopsis and many vegetables, condiment and oilseed crops. *B. rapa* is an ancestral species of *B. napus* and *B. juncea* (Nagaharu, 1935) and is mainly grown for leafy vegetables and healthy oil for human consumption in temperate regions of Canada, China, and Europe [16, 17]. It consists of highly diverse morphotypes, such as Bok Choy (ssp. *chinensis*), napa cabbage (spp. *perkinesis*), field mustard (spp. *oleifera*), Mizuna (ssp. *nipposinica*), turnip (ssp. *rapa*), and yellow sarson (ssp. *tricholaris*) [18]. Most of the *B. rapa* cultivars are highly prone to shattering and therefore it is pivotal to improve this trait in commercial cultivars by genetic interventions so that pods retain the seeds and can be harvested by machine without any yield loss.

There is a limited genetic variation for pod shatter resistance in accessions of *B. rapa* and its relatives [19–23] that is insufficient to circumvent pre-harvest and during mechanical harvesting seed loss. Several selection methods based on field observations, pan shaker, cantilever, random impact, rupture energy (RE), anatomical test and large replum area have been employed to assess genetic variation for pod shatter resistance in Brassica species [10, 24–29]. Among these methods, RE - a measure of pod strength has been commonly used to determine genetic variation in pod shatter resistance and inheritance studies in *B. rapa* [21], *B. carinata* [30–32], *B. juncea* [21] and *B. napus* [7, 33, 34].

Genetic analysis using biparental and genome-wide association studies (GWAS) revealed genome-wide QTLs, controlling pod shatter resistance in *B. napus*, *B. juncea* and *B. carinata* using the random impact test [35, 36] and RE measures [7, 30, 34]. These studies suggest that pod shatter resistance is controlled by complex genetics mediated by multiple genes having quantitative allelic effects, and environmental factors. In *B. rapa*, Mongkolporn et al. [20] reported the genetic control of pod shatter resistance due to two major recessive genes, referred to as *sh1* and *sh2*, which have a dominant epistasis effect in an F_2_ population derived from a cross between the parental lines DS-17-D (Indian origin) and Torch (Canadian cultivar). Both *sh1* and *sh2* genes were mapped with randomly amplified polymorphic markers. However, none of them has yet been mapped on the genetic linkage and physical maps of *B. rapa* and utilised for marker-assisted/genomic selection to improve pod shatter resistance in commercial cultivars of *B. rapa* and related species. Introgression of *B. rapa* genes into other *B* and *C* genome species is relatively straightforward and has been accomplished in several *B. napus* breeding programs, especially in China. For example, Raman et al., (2014) showed that the interspecific line derived from *B. napus/B. rapa* had up to 12-fold more RE compared to a shatter-prone *B. napus* variety. More recently, Raman et al. [34] identified seven QTL associated with RE on A02, A03, A05, A09 and C01 chromosomes in an F_2:3_ derived population from the cross between an interspecific breeding line BC95042 derived from *B. rapa/B. napus* and an advanced breeding line, BC95041.

In Arabidopsis, a set of master valve margin identity genes: *SHP1/SHP2*), *FUL* [15], *IND* [14], *ALC* [37], and *RPL* [38] have been identified that control pod dehiscence. In addition, plant hormonal orchestration also controls lignification and, hence, dehiscence [39–41]. However, the gene network has not been validated in Brassica crops due to the lack of an array of mutants and the complex polyploid nature of many *Brassica species*. Genetic mapping studies prioritised *AG*, *ABI3*, *ARF3*, *BP1*, *CEL6*, *FIL*, *FUL*, *GA2OX2*, *IND*, *LATE*, *LEUNIG*, *MAGL15*, *NST2*, *RPL*, *QRT2*, *RGA*, *SHP1*, *SPT* and *TCP10* candidate genes underlying QTL for pod shatter resistance with main and/or epistatic effects in *B. napus*,* B. juncea and B. carinata* [7, 21, 29–34, 36, 42, 43]. Mutant analysis studies also revealed that valve margin/DZ identity genes of *A. thaliana*: two MADS-box transcription factors *SHP1/SHP2*, *FUL*, a MADS-box gene in the valves and two bHLH family transcription factors, *IND* and *ALC* and *RPL* homeodomain gene in the replum regulate the pod shatter resistance in the close brassica relatives [44, 45]. To date, only two genes (*SHPI* and *NST2*) that confer resistance to pod shatter have been cloned in *B. napus* [42, 43]. However, a few studies have been carried out to understand the extent of genetic variation and its basis underlying pod shatter resistance in *B. rapa* [20, 21].

To understand the genetic variation in REamong *B. rapa* accessions, we used the pendulum machine test and identified QTL using a bulked segregant analysis (BSA) based QTL-seq approach [46–52]. Here, we revealed that natural variation for pod shatter resistance exists in *B. rapa* accession and is modulated by three QTL regions on the A06 and A09 chromosomes in an F_2_ population from a cross between ATC90153 and ATC91215 accessions. Alleles from ATC90153 contributed to shatter resistance and can be exploited in breeding programs to improve the level of shatter tolerance in commercial cultivars of *B. rapa* and closely related species.

## Materials and methods

### Plant materials

A diversity panel of 90 *B. rapa* accessions was accessed from the Australian Grains Genebank, Horsham, Australia (Table S1). Based on the contrasting pod shattering resistance (RE scores) of this diversity panel, we chose two lines: ATC90153 (shatter-resistant maternal line; RE = 2.65 mJ^(½)^), and ATC91215 (shatter-prone paternal line; RE = 1.08 mJ^(½)^) and made a cross between them to create F_1_ plants. An F_2_ population of 292 lines was raised by self-pollination of a single F_1_ plant and used for genetic analyses.

### Phenotyping for pod shatter resistance

The diversity panel comprising 90 accessions was raised during the 2012 and 2013 canola cropping seasons (April to December) with two replicates following an experimental design described previously [30]. Both parental lines and their F_2_ population of 292 plants were grown in the cropping season in 2015, following an experimental design in white plastic pots (Garden City Plastics, NSW, Australia) under birdcage conditions as described previously [34]. To estimate the reliability of variation in pod shatter resistance, both parental lines of an F_2_ population were replicated 10 times, and nine check lines (ATC92931, ATC95292, ATC90239 ATC95383 ATC95379 ATC95208, and ATC90310, BLN1990 and Surpass 400) were replicated 8 times in 2016 and each F_2_ line was unreplicated. All plant materials (384 lines) were grown in 32 rows × 12 ranges as per recommended agronomic practices at the Department of Primary Industries and Regional Development experiment site at Wagga Wagga (35.1026° S, 147.3655° E). Parental lines and their progenies showed segregation for flowering time (32–55 days), pod length and seed yield. All test lines were raised to maturity (BBCH scale 98), and then ten pods from each plant were collected in 50 mL conical plastic tubes to evaluate for shatter resistance as detailed in our previous studies [7, 30]. Pod shatter resistance was evaluated based on pod strength using RE as a proxy [25]. We measured pod length from each test sample using a scale excluding the size (length) of the ‘beak’ to adjust the pod’s position when the pendulum strikes. The RE data were square root transformed to normalize and further analysed using ASREML in R as described in Raman et al. [30]. The mean RE and pod length scores of five pods of each F_2_ plant were used for further genetic analysis. Anatomical features of valve margins were investigated by preparing hand sections from fresh pods (6–7 wk old) as described previously [7].

### DNA isolation

Leaf tissue of the birdcage-grown plants of diversity panel and F_2_ population was collected in 96 well boxes on ice and stored at -80 °C. Tissue was frozen in liquid nitrogen and ground with a mixer mill as described earlier [53]. High molecular weight genomic DNA was isolated using a standard cetyltrimethylammonium bromide method [54]. DNA purity and integrity were checked with agarose gel electrophoresis, and quantity was determined using a Qubit R 2.0 fluorometer (Life Technologies, Carlsbad, CA, USA). High-quality sample DNA (≥ 1.5 µg was used for library construction. Agilent R 2100 was used to assess the insert size.

### Genotyping of diversity and F2 population with DArT seq markers

The diversity panel of 90 accessions and the F_2_ population comprising 292 lines were genotyped with the genotyping-by-sequencing-based DArTseq marker approach [7] at the DArT P/L, University of Canberra, Australia. For genetic analysis, we considered only high-quality DArTseq markers, which included both SNP (single nucleotide polymorphism) and in-silico (presence-absence) markers, having BLAST alignments (E-value: 5e^-5^) and minimum sequence identity of 90% with the reference *B. rapa* genome version 3.5 [55].

### Genetic relatedness among *B. rapa* accessions

High-quality DArTseq markers having a call rate of ≥ 90%, ≤ 5% of missing data and a minor allele frequency (MAF) of > 0.05 were used for cluster and multi-dimensional scale analysis. The software PRIMER 6 [56] was used for hierarchical cluster analysis based on Euclidian distance. Principal coordinate analysis was performed to understand the global diversity among accessions.

### Whole genome resequencing and QTL-seq analysis

For the QTL-seq approach, an equal amount of DNA from ten F_2_ plants representing low RE (< 1.41 mJ^(½)^) and high RE (> 2.34 mJ^(½)^) was combined to form the bulk-sensitive pool (BS), and bulk-resistant pool (BR) respectively. In addition, we sequenced the parental lines of the F_2_ population using four µg of high-molecular-weight genomic DNA. Pair-end libraries with an insert size of 150 bp were prepared using the pair-end DNA sample prep kit (Illumina, San Diego, CA, USA). The sequence data of two parental and bulked pools were generated by the Illumina R HiSeq 2500 platform at Novogene (Schengen, China, http://www.novogene.com). Low-quality paired reads (> 50% based on having Phred quality ≤ 20) were trimmed to ensure the reads were reliable without artificial bias. The high-quality clean reads from two DNA bulks and parental lines were then used to align against the reference genome of *B. rapa* version 3.5 using SAMtools software [57]. SNPs or insertion/deletion mutations (Indels) were called from BAM files using the GATK version 3.7 software. These variants were annotated according to GFF3 files aligned with the *B. rapa* reference genome version 3.5 by ANNOVAR [58].

Association analysis based on the difference in the SNP index of the two pools was calculated as △ (SNP index) as described previously [47]. The △ SNP index of each polymorphic site was calculated by subtracting the SNP index of the BR pool from the BS pool. The candidate regions were narrowed down to windows with significant *P*-values at a 99% confidence level for mapping major QTLs. To identify QTLs with minor effect, 584 candidate SNPs with SNP-index close to 0 or 1 (≥ 0.8 in the BR pool and ≤ 0.2 in the BS pool) were selected and annotated by ANNOVAR. Candidate SNPs were annotated as stop loss, stop gain and other non-synonymous mutations at the coding region, and those with splicing-related mutations were highlighted as candidates. Genes with SNPs in the promoter region before starting codon ATG (≤ 1 kb) in their corresponding alleles were also selected as candidate genes. All raw sequencing data generated in this study were deposited in the NCBI SRA under PRJNA1209117.

### Map construction and QTL identification for pod shatter resistance

The linkage map of the F_2_ population was constructed using DArT P/L’s OCD MAPPING program [59], as described previously [30]. The association between markers and RE was tested using single-marker linear marker regression, haplotype trend regression (HTR), and multi-locus marker regression without accounting for kinship and population structure, typically used for GWAS in structured populations with historical recombination history. We applied the additive model with full scan permutation with 1,000 iterations for the genome scan and false discovery rate (0.05). These thresholds were used to identify significant marker associations. Haplotype block (HB) analysis and HTR were performed as detailed in our previous study [32] using the SVS package (Golden Helix, Bozeman, USA). Manhattan plots were generated in the SVS package (Golden Helix, Bozeman, USA).

### Prediction of candidate genes

The sequences of DArTseq markers and candidate genes for pod shatter resistance were searched in the *B. rapa* reference genome version 3.5 annotations and released in BRAD (www.brassicadb.cn) using BLASTn software [60]. Based on the sequencing data of parental lines, sequences of the predicted genes within the A06 (7.84–25.1 Mb) and A09 (38.2 to 38.8 Mb) QTL regions were compared for polymorphisms between the parents.

## Results

### Genetic variation in rupture energy

**Fig. 1:**
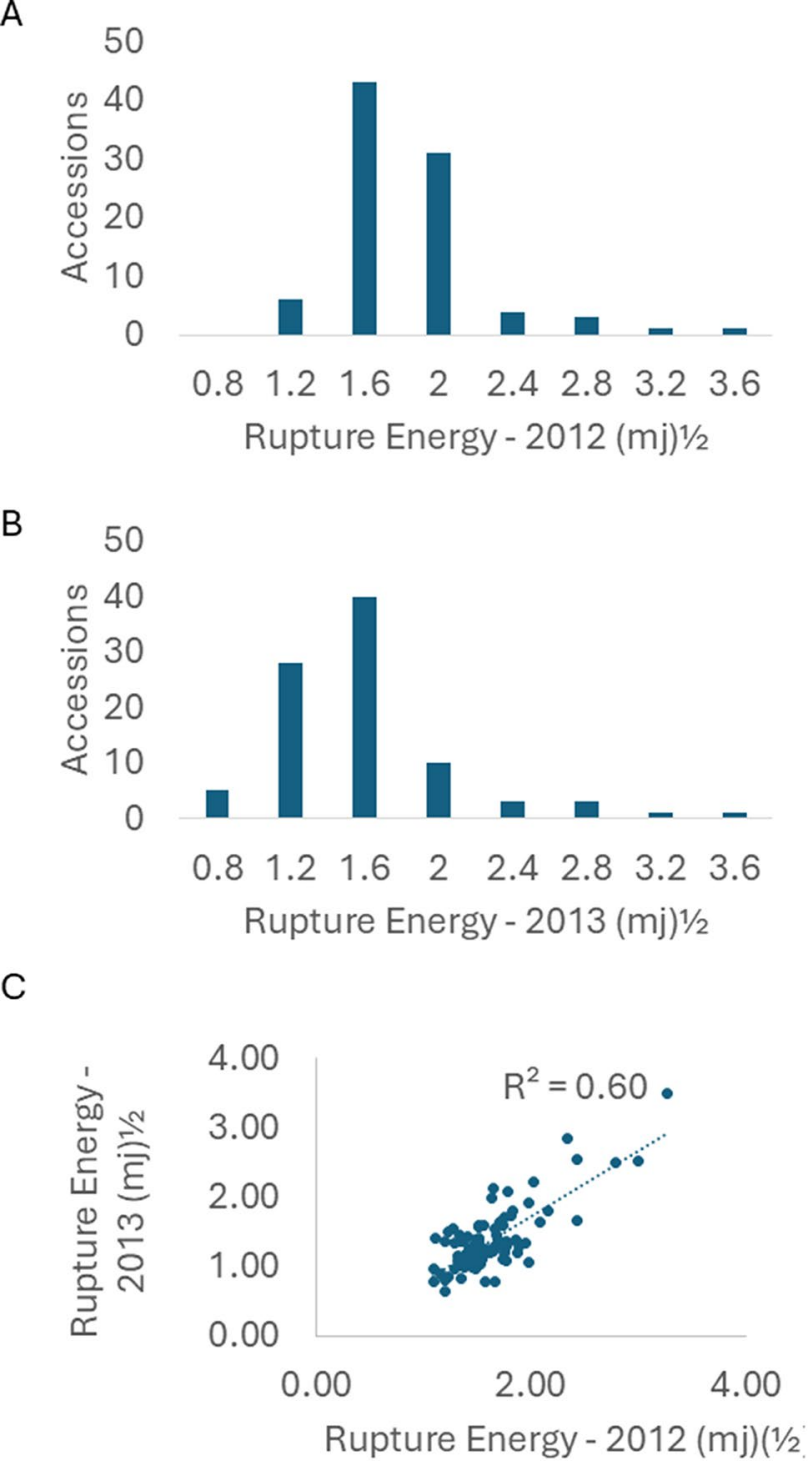
Frequency distribution (**A**,** B**) and correlations of rupture energy (expressed as square root means) of 90 *Brassica rapa* accessions (**C**), representing different ssp., grown at the Wagga Wagga Agricultural Institute across two growing seasons (2012 and 2013)

A panel of 90 accessions of *B. rapa* was evaluated for pod shatter resistance, measured as RE, across two seasons (2012 and 2013). This panel represents nine subspecies per Australian Grains Genebank collection passport data (Table S1). A considerable genetic variation in pod RE ranged from 1.09 to 3.27 mJ^(½)^ in 2012, and 0.63 and 3.49 mJ^(½)^ in 2013 was observed (Table S2, Fig. 1). The RE of ‘check’ *B. napus* lines, BLN2762 and Surpass400 varied from 1.47 to 1.68 mJ^(½)^ in 2012 and 1.86 to 2.07 mJ^(½)^ in 2013 environment. Eight *B. rapa* accessions, ATC92931, ATC95292, ATC90153, ATC90239 ATC95383 ATC95379 ATC95208, and ATC90310, had higher RE than the *B. napus* checks (Table S2). A strong positive correlation (*r* = 0.77) among accessions evaluated across the 2012 and 2013 growing environments was observed (Fig. 1C), indicating that RE is genetically controlled and stable across two test environments.

### Cluster and principal coordinate analyses of *B. rapa* accessions

**Fig. 2:**
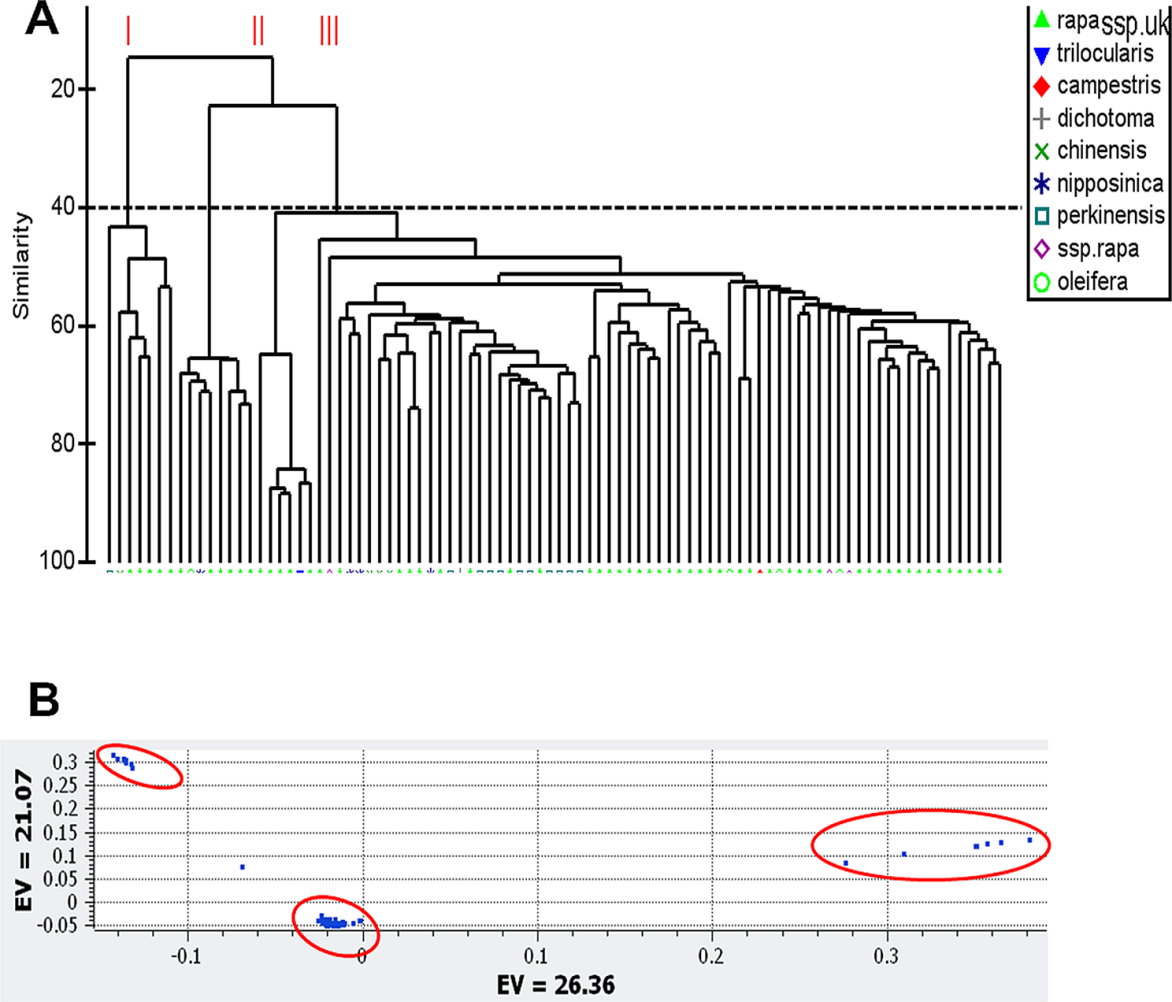
Cluster and principal coordinate analyses of 90 *Brassica rapa* accessions, representing different subspecies as catalogued in the Australian Grain Genebank, Horsham. **A**: A phylogenetic tree based on 10,420 markers was drawn using Primer 7 using the Jaccard coefficient. Based on a 40% resemblance, three clades labelled as I-III were revealed. UK: unknown. Parental lines of the F_2_ mapping population are underlined in red. **B**: Principal component analysis of 90 *B. rapa* accessions showing grouping into three clades (circled in red colour). EV: Eigenvector

To investigate the genetic relationships between *subspecies* of *B. rapa*, we performed cluster analysis using 10,324 high-quality DArTseq markers. This analysis grouped accessions in three (I to III) clades (Fig. 2A). The principal component revealed that the first two eigenvectors accounted for 26.36% and 21.07% of the molecular variation, respectively (Fig. 2B).

**Fig. 3:**
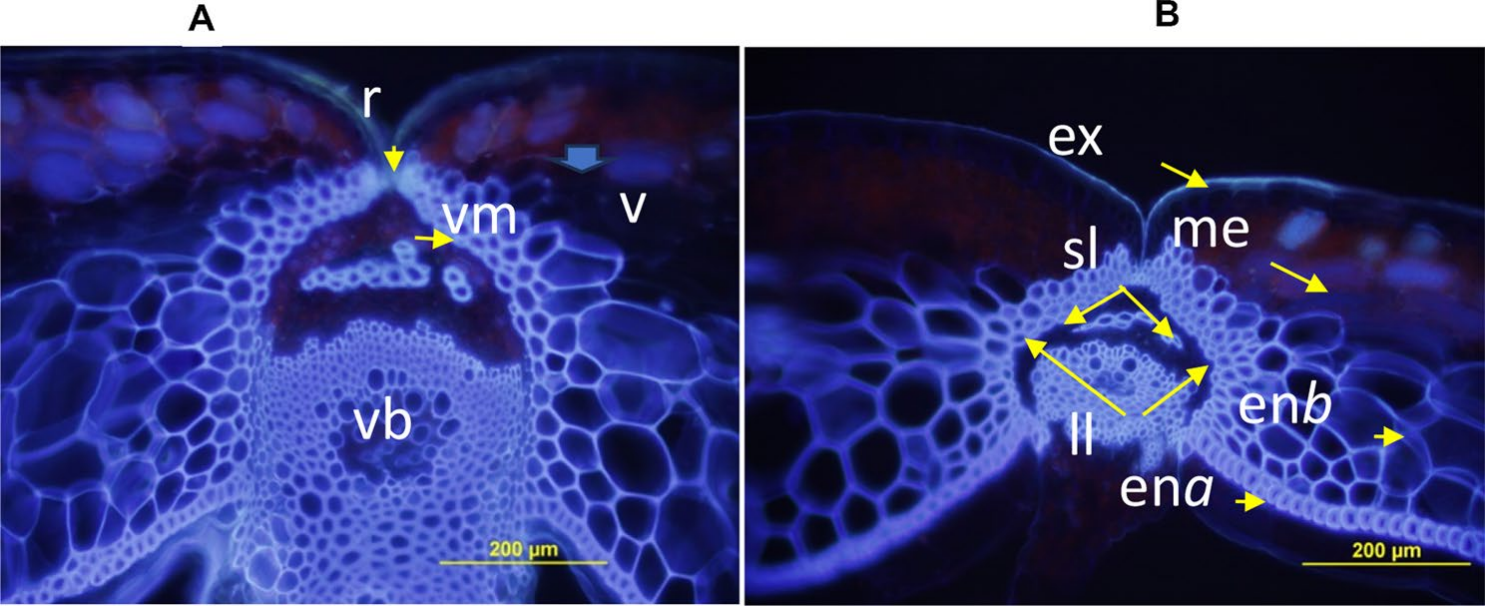
Anatomical features of the developing pods in the parental lines of the F_2_ population from the ATC90153/ATC91215 grown in 2016. (**A**) ATC90153: High RE (shatter resistant) and (**B**) ATC91215: low RE (shatter prone). The transverse section of ATC91215 shows well-developed DZ, whereas ATC90153 shows almost no DZ differentiation after 5 weeks of pollination en*a*; endocarp *a* layer; en*b*; endocarp *b* layer; ex: exocarp; ll: lignification layer; me: mesocarp; r: replum; sl: separation layer; v: valve; vm: valve margin; and vb: vascular bundles: Bar: 200 μm

We chose two accessions, ATC90153 and ATC91215, based on their RE scores and molecular polymorphisms (Table S2, Fig. 2) and generated an F_2_ population for genetic analysis. The anatomical pod structure of both parental lines also showed differences in lignification (ll) and separation layers (sl) at 30 days of pollination between the valve (v) and replum (r), essential for DZ formation (Fig. 3). Similar anatomical differences were reported in other pod shatter-resistant and shatter-prone natural, mutant and wild-type accessions of Brassica species and Arabidopsis [9, 61].

**Fig. 4:**
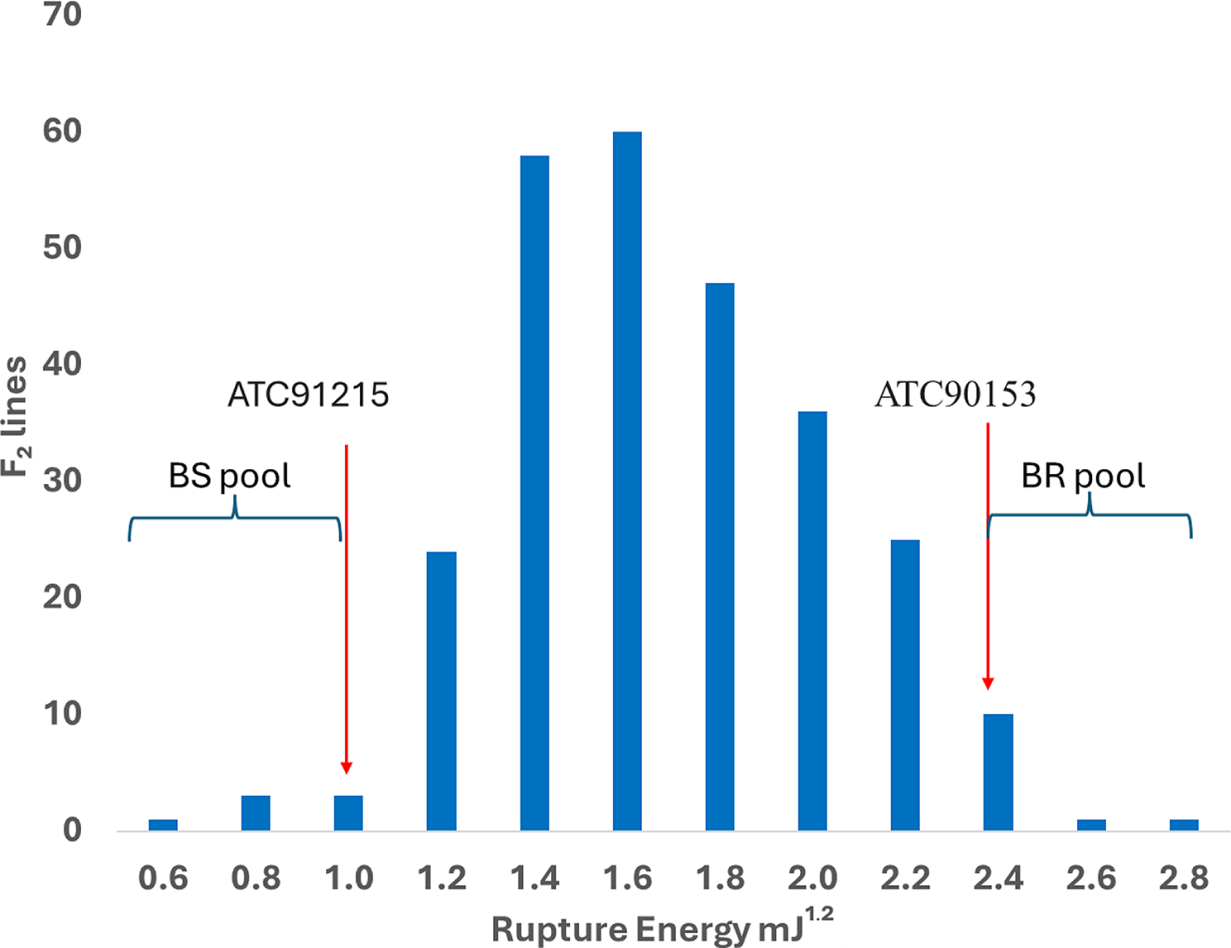
Frequency distribution of rupture energy (RE) scores in the F_2_ segregating population comprising 292 individuals, derived from ATC90153/ATC91215. The average RE scores (square root means) of the parental lines, ATC90153 and ATC91215, are indicated by red arrows. BR: Bulked shatter-resistant pool and BS: Bulked shatter-sensitive pool

To investigate the genetic control of pod shatter resistance, 292 F_2_ lines and the parental lines ATC90513 and ATC91215 were assessed for variation in RE under birdcage conditions. The RE scores of the pod-shatter-resistant line, ATC90513, were higher 2.34 mJ^**½**,^ than those of the pod-shatter-prone line, ATC91215 (1.08 mJ^**½**^). The frequency distributions of RE in the F_2_ population showed a normal distribution, ranging from 0.58 to 2.71 mJ^**½**,^ with a mean score of 1.59 mJ^**½**^ (Fig. 4). Seven F_2_ lines showed transgressive segregation; their RE scores were more towards the pod-shatter-prone maternal parent. We further found that pod length and RE scores were positively correlated (*r* = 0.44, Figure S1).

### Whole genome resequencing and QTL-seq analysis

QTL-seq analysis based on whole genome resequencing was performed to identify loci for pod shatter resistance. Sequencing of the genomic DNA from both parental lines, ATC90153 (high RE) and ATC91215 (low RE), and the two pools (BR pool and BS pool) resulted in 0.2 billion clean reads. Most of the reads obtained were high quality, with Q30 ≥ 91.36% (Table 1). Cleaned reads were mapped to the *B. rapa* reference genome v3.5, and analysis showed that at least 83.67% of them had a minimum of 4× coverage (Table S3).

**Table 1:** Descriptive statistics of resequencing data of both parental lines of an F_2_ population from ATC90153 and ATC91215 and the bulked resistant (BR) and sensitive (BS) pools

#Sample	Raw bases (bp)	Clean bases (bp)	Effective rate (%)	Error rate (%)	Q20 (%)	Q30 (%)	GC content (%)
ATC90153	51,917,846,100	51,001,074,300	98.25	0.01	96.29	91.53	37.31
ATC91215	53,174,812,800	52,307,058,300	98.38	0.01	96.22	91.36	36.53
BR pool	50,543,745,000	49,532,344,800	98	0.01	96.53	92.04	36.07
BS pool	49,875,930,600	48,911,737,800	98.06	0.01	96.31	91.70	35.89
Mean	51,378,083,625	50,438,053,800	98.17	0.01	96.34	91.66	36.45

**Fig. 5:**
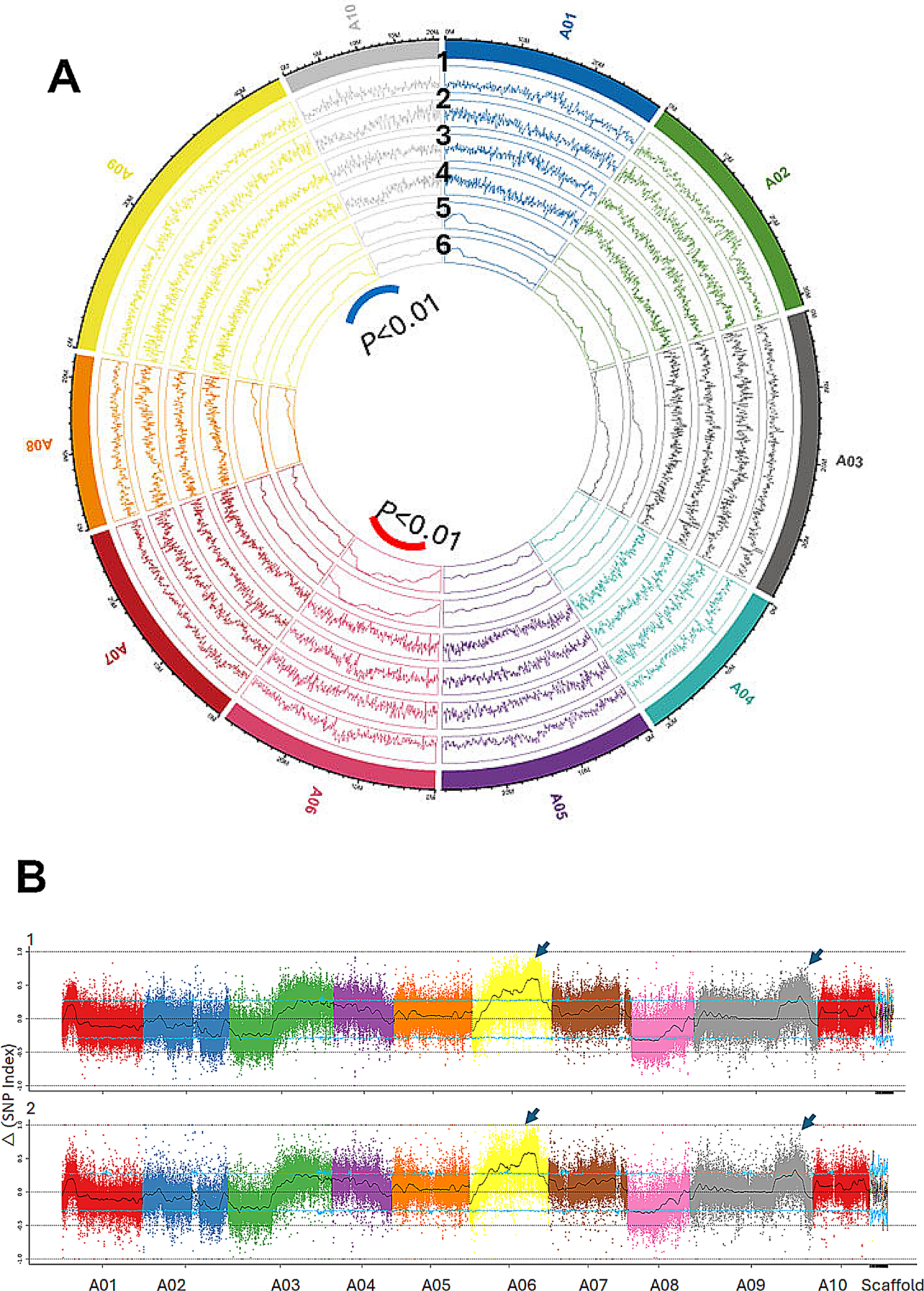
Distribution △SNP (**A**) and △InDel (**B**) indices along *B. rapa* reference genome v3.5. **A**: Circos plot showing the distribution of SNP (1) in ATC90153, and (2) ATC91215; InDels in (3) ATC90153 and (4) ATC91215; (5) △ SNP in the bulked pooled pod shatter-resistant and shatter-prone pooled samples and (6): △ InDel in the bulked pooled pod shatter resistant and shatter prone pooled samples. **B**. QTL identified using △SNP (upper panel) and △InDel (lower panel). QTL peaks are marked with arrows. The *x*-axis represents the chromosome position, and the *y*-axis indicates the △(SNP-index). The SNP/InDEL index of each polymorphic site was calculated by subtracting the ‘BR pool’ SNP index from the ‘BS pool’

Sequence variants in the form of SNP and InDels were mapped to the reference *B. rapa* genome assembly. SNP and InDel density varied from 3,760,348 to 5,393,671 and 869,669 to 1,310,804 in the parental lines and bulked pooled samples, respectively and had a genome-wide coverage (Table S4, Fig. 5A). There was a high proportion (1.35 to 1.37×) of transition SNP as compared to transversion among samples. Among SNPs, C: G > T: A was more frequent, followed by T: A > C: G. As expected, there was a high rate of heterozygosity in the bulked pools compared to parental lines (Table S5). The InDel frequency among samples ranged from 50.9 to 51.6 and was inversely related to length (1 to 20 bp). Chromosome A06 had the maximum density compared to other chromosomes. Annotation analysis identified 3,760,348 to 3,857,820 SNPs and 869,669 to 882,876 InDels in the upstream, exonic and downstream sequences in bulked pooled samples (Table S6). The SNP/InDel indices of the two DNA pools were calculated for each variant separately (Table S7A-C). The ΔSNP/InDEL index was calculated and plotted to the genome position by combining the information of the SNP index in the BR pool and BS pool (Fig. 5B). This analysis showed the genomic regions associated with RE were located on chromosomes A06 and A09. The 225 SNP variants showed a large effect on pod shatter resistance; of them, 19 SNPs were in downstream sequences on A06, 41 non-synonymous SNPs (39 on A06, one each on A02, and A03 chromosomes), and 165 SNPs were present in upstream sequences on chromosomes A06 and A09 (Table S7D). In addition, 195 large effects InDels were identified in downstream and upstream CDS and frameshift, stop-gain, and splice variants (Table S7E). Both datasets on SNPs and InDels suggested that chromosomes A06 and A09 harbour genomic regions of interest. We chose peak regions on both chromosomes above the threshold value of 0.2 to select the genomic region for the pod shatter resistance. With a 99% significance level, a total of 24 variants were prioritised underlying 7.35 Mb genomic regions on chromosome A06 (20 sequence variants in 7,345,001–14,706,001 bp of the reference sequence) and 0.6 Mb on chromosome A09 (4 sequence variants within 38,243,001 to 38,844,001 bp) for pod shatter resistance (Table S7F-H). In addition, 25 variants mapped to the unanchored scaffolds were also associated with pod shatter resistance in the test population of *B. rapa* (Table S8). This analysis showed that two genomic regions on A06 and A09 modulate RE in the test *B. rapa* F_2_ population.

### Validation of identified QTL-seq genomic regions using classical QTL analysis

To verify the genomic region predicted from QTL-seq, traditional QTL mapping was performed in an F_2_ population from ATC90153/ATC91215. DArTseq analysis revealed 50,723 SNPs and in silico polymorphisms segregating in the F_2_ population (Table S9A). Using the 23,274 high-quality markers, we constructed a linkage map of the ATC90153/ATC91215 population (Table 2). The markers were distributed across all ten chromosomes of *B. rapa* and covered a 909.75 cM distance. The marker density varied from 21.82 (A05) to 31.14 (A09)/ cM, with an average density of 25.58 markers/cM. Given that the total genome of *B. rapa* is approximately 450 Mb, this equates to 494.64 kb/cM. This genetic linkage map was further used for the QTL identification.

**Table 2:** Summary of segregating markers and their coverage on the linkage genetic map of the F_2_ population derived from the ATC90153/ATC91215 of *B. rapa*. cM: Centimorgan

Chromosome	Mapped markers (no.)	Map length (cM)	Average marker density/cM
A01	2,485	109.07	22.78
A02	2,130	78.58	27.11
A03	3,283	136.35	24.08
A04	1,442	50.85	28.36
A05	1,984	90.94	21.82
A06	2,326	104.12	22.34
A07	1,863	76.12	24.48
A08	1,864	63.74	29.24
A09	4,187	134.46	31.14
A10	1,710	65.51	26.10
**Total**	**23**,**274**	**909.75**	**25.58**

**Fig. 6:**
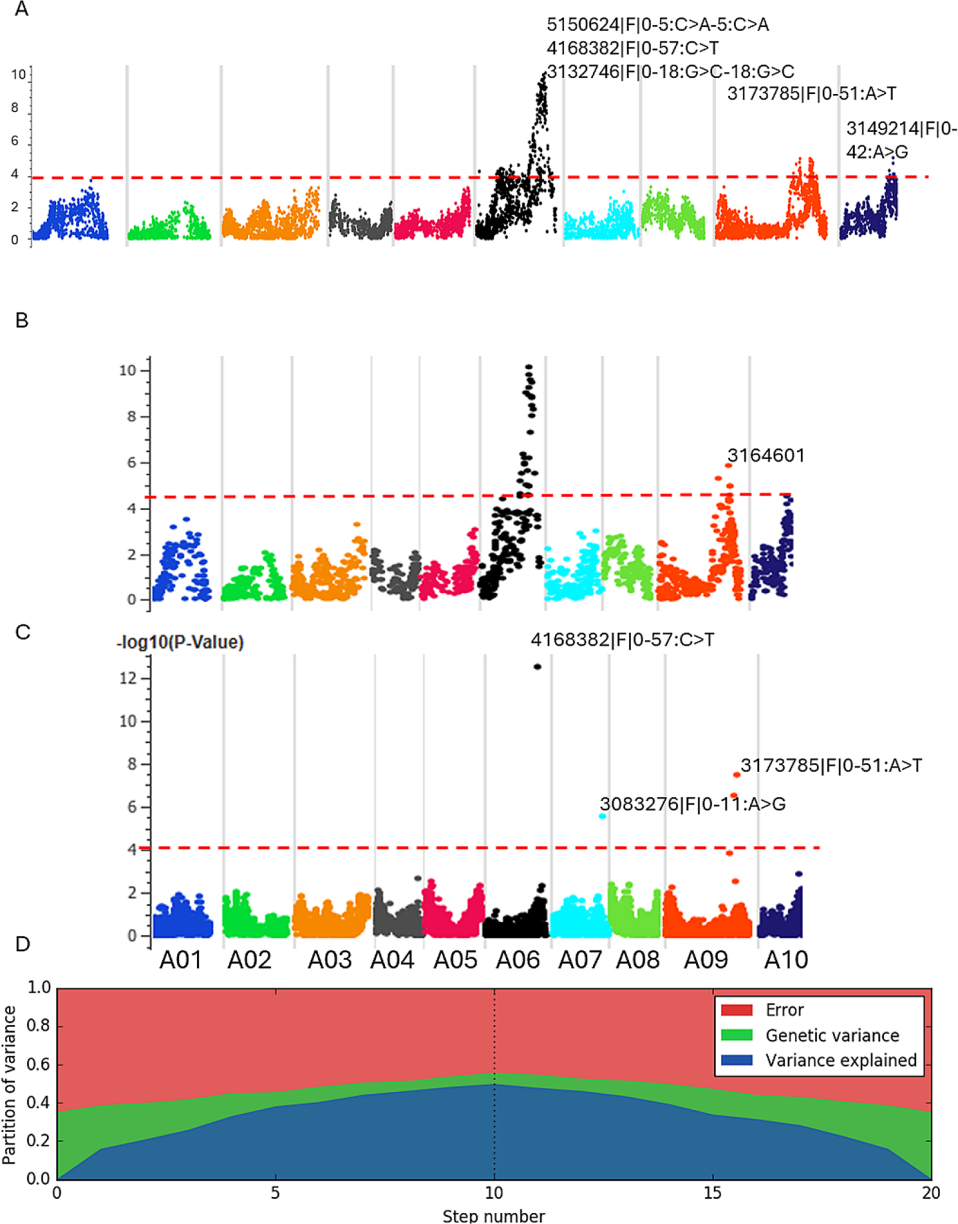
Genetic mapping of pod shatter resistance in *Brassica rapa* population derived from a single F_1_ plant of ATC90153/ATC91215. **A**. Manhattan plots showing a single marker linear regression for pod shatter resistance (RE). **B**. Haplotype trend regression identifies significant associations for shatter resistance. **C**. Multi-locus mixed model (additive) identifies two significant SNPs at the Bonferroni-corrected threshold and a False discovery rate of 0.05. **D**. The partition of the variance plot at each step (10 forward and 10 backward) into variance is explained by the SNPs in the model (blue), the kinship among F_2_ lines (green), and the noise (red). Highly significant markers are also shown; markers depicted with > denote DArTseq SNP markers, and those without > symbol denote in silico DArT. The threshold LOD value for the trait-marker association is shown as a dashed line

A genome scan using single-locus regression showed that three genomic regions on chromosomes A06, A09 and A10 were significantly associated with pod RE (Fig. 6A, Table S9B). HTR identified a suite of 31 markers associated with RE; of them, 27 were mapped on chromosome A06, and four were mapped to chromosome A09. None of the other markers met the genome-wide Bonferroni and false discovery rate (FDR) thresholds used in this study. The top 18 HB on A06 had LOD scores ranging from 5.89 to 10.14 (Table S9C). With stepwise multi-locus-based regression, we identified five marker associations for RE on chromosomes A01, A06, A07 and A09 with -*log*10(*P*) ranging from 5.14 to 12.66 (Table S9D). The markers 100137462 on A01, and 4168382|F|0–57:C > T-57:C > T (-*log*10(*P*) = 7.36) on A06 (-*log*10(*P*) = 12.66), showed distorted segregation. The other two highly significant markers 3173785|F|0–51:A > T-51:A > T on A09 (*-log*10(P) = 9.33) and 3105087|F|0–23:C > A-23:C > A on A09 associated with RE revealed normal monogenic segregation ratio (*χ2*: 4.08 to 4.38, *P* = 0.111 to 0.129) (Table S9d). The 3083276|F|0–11:A > G-11:A > G on A07 did not meet the Bonferroni threshold (Table S9d). In this population, we detected three genomic regions for pod length on A02, A06 and A07, A06 locus mapped to the QTL regions for pod shatter resistance (Table S10). However, the other two QTLs on A02 and A07 were unique for pod length. These findings suggest that pod RE and length are partly related traits in this population (Figure S2).

### Identification and functional annotation of candidate genes for pod shatter resistance

**Fig. 7:**
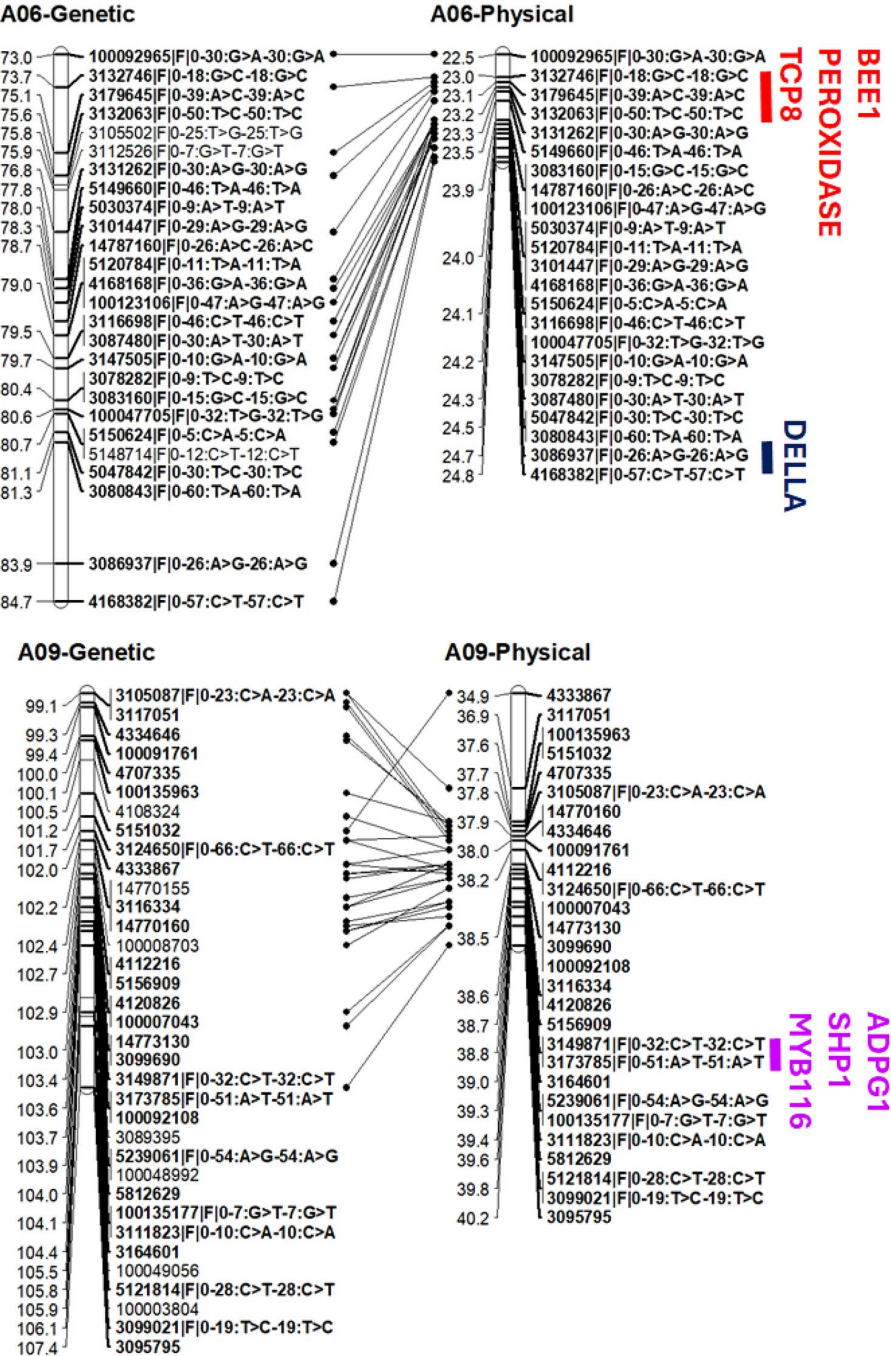
Putative candidate genes that mapped near the significant markers associated with pod shatter resistance in the *Brassica rapa* population derived from a single F_1_ plant of ATC90153/ATC91215. Positions of DArTseq markers and candidate genes were obtained by aligning sequences with the reference genome sequence assembly of *B. rapa* cv. Chiifu (version 3.5). Genetic (cM) and physical positions (x 10^5^ bp) are shown on the left side of the map. Markers that were mapped on both genetic linkage and physical (reference genome) are joined with solid black lines

DArTseq markers were assigned the physical positions based on their sequence identities with the reference genomes of *B. rapa*. QTL mapping results were consistent with thos obtained from Δ (SNP-index and InDel index) analyses of QTL-seq data, supporting that genomic regions for RE are located on A06, 7.35 Mb − 25.1 Mb and A09 (38.2 to 38.8 Mb). We further searched the sequence identities with the *priori* candidate genes involved in the regulation of pod dehiscence in Arabidopsis (*AG*,* AP2*,* SHP1/SHP2*,* IND*,* MAN7*,* ALC*,* RPL*,* ADPG1/ADPG2*,* JAG*,* LATE*,* NST1/NST2/NST3*,* FIL*,* YAB3*,* DELLA*,* TCP8*,* SPT*,* PID*, and *WAG2*) with the reference genomes of *B. rapa*. Of them, four candidates; Brassinosteriod signalling gene, *BEE1* (BraA06g014130), *PEROXIDASE* (BraA06g018390) and *TCP8* (BraA06g01190) on the proximal end, and *DELLA* on the distal end of chromosome A06; and *SHP1* (*SHATTERPROOF 1*, BraA09g050860), *ADPG1* (BraA09g050000) and *MYB116* on chromosome A09 homologues were located near the QTL regions (Fig. 7). However, the closest candidate *SHP1* was located within 0.5 to 1.2 Mb from the significant SNP markers on chromosome A09 identified using QTL and **Δ**SNP approaches. Further research is required to establish whether these candidates modulate pod shatter resistance via fine mapping and functional gene analyses.

## Discussion

### Genetic variation for pod shatter resistance in *B. rapa*

Domestication of crops by intentional (human selection) and unintentional (environmental/evolutionary selection) shaped the existing genetic variation in crop plants. Seed shattering is one of the hallmark domestication traits subjected to the selection of major food crops such as wheat, barley, maize, rice, and soybean. As a result, these crops show a loss of this trait in many traditional landraces and contemporary varieties while transitioning from wild to facilitate harvesting [62]. However, resistance to seed (pod) shattering in Brassica crops has probably ‘escaped’ from intensive selection during domestication [7] that occurred several thousand years ago. As a result, none of the diploid ancestral species (*B. rapa*, *B. oleracea*, and *B. nigra*) and cultivated amphidiploid species that arose from interspecific hybridisation events between diploid ancestral species [16] are indehiscent or completely resistant to pod shattering.

Given that limited research has been conducted to access genetic variation from *B. rapa* for breeding pod shatter-resistant *B. rapa*, *B. juncea* and *B. napus* cultivars, this investigation evaluated 90 accessions representing different morphotypes, e.g. vegetable and oilseed. We found that most *B. rapa* accessions evaluated in this study are prone to pod shatter (Table S2) including those evaluated in previous studies [20–22] compared to pod shatter-resistant accessions of *B. carinata* [30], supporting the hypothesis that this trait largely escaped from selection during the domestication of *B. rapa and B. napus*. We chose two accessions, ATC90153 and ATC91215 and developed an intercross population for genetic analysis. The resistant accession, ATC90153, lacked a well-developed abscission layer, whereas the sensitive line, ATC91215, had lignification of the junction of the valve and the replum (Fig. 3). These findings are consistent with the previous studies, which revealed less lignification in shatter-resistant/intermediate-resistant lines than in shatter-prone lines [7, 61].

### Multiple QTL modulate variation for pod shatter resistance in the *B. rapa* population

Genetic analysis based on single marker regression, interval mapping, and multiple QTL mapping compositive mapping approaches are used in structured populations. However, most packages do not accommodate large sets of markers (several thousand markers utilised in this study). Herein, we used the SVS-Golden helix and SNP index pipelines to identify trait-marker associations. As expected, the multi-locus model performed better than the single-locus regression; the latter model showed a substantial inflation of LOD scores [63]. QTL-seq based on BSA (SNP and InDEL index) and QTL mapping approaches enabled us to identify at least two QTL for pod shatter resistance on chromosomes A06 and A09. We found that several markers associated with RE (SNPs and in-silico DArT) on the A06 chromosome showed segregation distortion. This study does not yet establish whether this is due to biological factors. Further research is required to prove this hypothesis. We showed that shattering resistance in ATC90153 accession is governed by three QTL suggesting that multiple genes on chromosomes A06 and A09 are responsible for trait variation. We revealed that a large genomic region (17 Mb) showed a significant association with pod shatter resistance. Fine mapping is required to resolve genetic factors underlying this trait variation.

Previous studies have shown that pod shatter resistance in *B. rapa* is due to two major genes, *sh1* and *sh2*, with dominant epistasis [20, 22]. These recessive genes were tagged with RAPD markers (RAC-3_900_, RX-7_1000_ and SAC-20_1300_). However, these markers have not yet been anchored to *B. rapa* chromosomes/genome. Therefore, it isn’t easy to ascertain whether the loci identified in this study correspond to *sh1* and *sh2* genes. Kaur et al., [21] performed GWAS and candidate genes analyses of pod shatter resistance in 90 accessions of *B. rapa*, 24 of *B. nigra* and 124 of *B. juncea*. The authors identified marker-trait associations for RE in *B. juncea*; however, no significant association for RE was identified in *B. rapa*. Here, we localised three significant loci for RE to chromosomes A06 and A09. These genomic regions may represent the QTL on A06 and A09 reported in *B. napus* [7, 36]. The A06 QTL was detected repeatedly across environments in the diversity panel of 143 rapeseed accessions and DH and inter-mated F_2_ populations and mapped near the GIBBERELLIN-3-OXIDASE gene [36]. On A09, three candidate genes associated with pod shatter resistance have been identified. For instance, *SHP1* and *NST2* genes control pod shatter resistance at *qSR1 9.1* and *qRT1.9.2* in the R1/R2 population [42, 43]. Another candidate gene, *FUL*, has been mapped near the QTL for pod shatter resistance on the A09 locus in *B. napus* [36] and an interspecific population of *B. rapa/B. napus* [7, 34] and in two populations of *B. carinata* [30]. Overall, available QTL and GWAS data suggest several alleles are associated with pod shatter resistance in cultivated germplasm and may have been selected in the brassica breeding programs. Evaluation of the predicted genes within the candidate region provided a list of potential candidate genes such as *IND* and *SHP1/SHP2*. Both genes are known for their role in the regulation of pod dehiscence in *A. thaliana* [15, 64] and *B. napus* [42, 65].

### Comparative analysis of genetic mapping approaches

Both approaches based on QTL-seq and classical QTL approaches were suitable for identifying genomic regions for pod shatter resistance in *B. rapa*. QTL-seq using the BSA approach using 10 resistant and 10 shatter-prone lines and whole genome re-sequencing of those selected individuals also detected the same genomic regions on A06 and A09 as detected by classical QTL mapping (Fig. 6). In recent years, whole–genome sequencing and bulked segregant analysis (BSA/QTL-seq) have been widely used in the gene mapping and molecular marker development of key crops, including maize [51], rice [47], cotton [66], rapeseed and Ethiopian mustard [31, 52, 67, 68]. Our results showed that genetic loci with moderate allelic effects could be identified using the BSA-resequencing approach, as shown here. In addition, this analysis resolves polymorphisms at the nucleotide level and is, hence, more suited for detecting allelic variations in genetic, evolution, and adaptation studies.

### Application in Brassica breeding

Pod shatter tolerance is a highly desirable trait for reducing harvest losses and achieving stable yield. *B. rapa* is generally grown in the Indian subcontinent and South Asia for food and healthy oil. However, it is not grown commercially as an oilseed crop in other parts of the world except as a source of vegetables (Bok Choy, Choy sum). In this study, we have identified a superior source of pod shatter resistance in *B. rapa* and mapped the three QTL on A06 and A09 using molecular markers. The closely linked molecular markers (LOD score of 6 to 12) can be used for improving pod shatter resistance in *B. rapa* and for genomic-assisted breeding of close relatives, especially *B. napus* and *B. juncea*. Interspecific hybridisation between *B. napus* and *B. rapa*/*B. juncea* is more compatible and less challenging than *B. carinata*. The QTL on A06 and A09 can be combined with other QTL identified in previous studies and therefore by accumulating favourable alleles, pod shatter resistance can be possibly improved so that yield losses can be minimised at the farm level.

## Conclusions

This study describes the extent of genetic variation and provides an understanding of the genetic basis of pod shatter resistance in *B. rapa*. The pod shatter resistance accessions and QTL on chromosomes A06 and A09 would enable breeding for pod shatter resistance in Brassica crops.

## Electronic supplementary material

Below is the link to the electronic supplementary material.

Supplementary Material 1

Supplementary Material 2

Supplementary Material 3

Supplementary Material 4

Supplementary Material 5

Supplementary Material 6

Supplementary Material 7

Supplementary Material 8

Supplementary Material 9

Supplementary Material 10

Supplementary Material 11

## Data Availability

All raw sequencing data generated in this study has been deposited in the NCBI (https://www.ncbi.nlm.nih.gov/sra/PRJNA1209117). The request for material should be addressed to Harsh Raman.

## Abbreviations

ADPG1: ARABIDOPSIS DEHISCENCE ZONE POLYGALACTURONASE 1
ALC: ALCATRAZ
BSA: Bulked segregant analysis
BEE1: Brassinosteroid signalling gene
cM: CentiMorgan
DZ: Dehiscence zone formation
FUL: FRUITFUL
GWAS: Genome-wide association studies
HB: Haplotype block
HTR: Haplotype trend regression
IND: INDEHISCENT
Kb: Killobase
LOD: Logarithm of the likelihood ratio
Mb: Megabase
NST: NAC Secondary Wall Thickening Promoting Factor
RPL: REPLUMLESS
QTL: Quantitative trait loci
RE: Rupture energy
SNP: Single nucleotide polymorphism
SHP1: SHATTERPROOF 1
QTL-Seq: Quantitative trait locus sequencing
TCP8: TEOSINTE BRANCHED1/CYCLOIDEA/PROLIFERATING CELL FACTOR 8
WGR: Whole genome resequencing
