# Supplementary material for: Identification and validation of genomic regions for pod shatter resistance in Brassica rapa using QTL-seq and traditional QTL mapping
Source: BMC Plant Biol. 2025 Feb 10;25:175. doi: 10.1186/s12870-025-06155-z (PMC11808946; doi:10.1186/s12870-025-06155-z)
Supplement: Supplementary file 1 — Supplementary Material 1 [file 12870_2025_6155_MOESM1_ESM.docx]

Figure S1. The relationship between the pod length and pod shatter resistance, measured as rupture energy (mJ) in the F_2_ population derived from ATC91215 and ATC90153.

Figure S2. **Genetic mapping of pod length in *Brassica rapa* population derived from a single F_1_ plant of ATC90153/ATC91215.** A. Manhattan plots showing a single marker linear regression for pod length. Multi-locus mixed model (additive) identifies three significant DArTseq-SNPs at the Bonferroni-corrected threshold and a False discovery rate of 0.05. D. The partition of the variance plot at each step (10 forward and ten backward) into variance is explained by the SNPs in the model (blue), the kinship among F_2_ lines (green), and the noise (red). The threshold LOD values for the trait-marker association are shown as a dashed line.


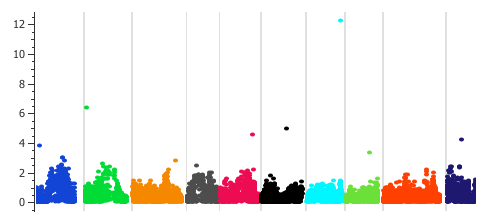

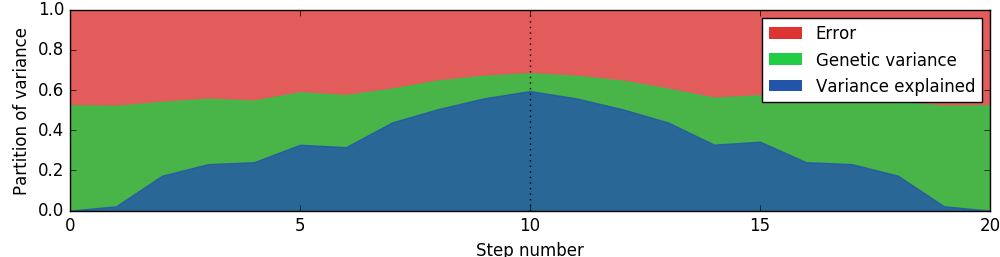


3080981|F|0-30:C>T-30:C>T

3106183|F|0-61:T>A-61:T>A

4335545|F|0-28:C>T-28:C>T

A01 A02 A03 A04 A05 A06 A07 A08 A09 A10
